# New Elements of Operative Surgery

**Published:** 1847-04

**Authors:** 


*New Elements of Operative Surgery:* By ALF. A. S. M. VELPEAU. Accompanied with an Atlas of twenty-two plates. Translated by P. S. TOWNSHEND, M. D., under the supervision of VALENTINE MOTT, M. D., etc., Vol. III. New York: Samuel S. & Wm. Wood, 1846. pp. 1162, 8 vo. (From the Publishers.)

We have, some time since, acknowledged the receipt of the second volume of this work with the promise of a further notice at a future time. The third, with the atlas, completing the work, is now before us, but we are still unable to do more than announce its appearance. Indeed, we must despair of being able to give an analysis of its contents. It is a work which embraces almost every known or imagined operation of early or late times, and is indispensable as a work of reference to surgeons. While the additions of the American editor and translator embrace near 1500 pages, there is little of American surgery recorded there except the operations and report of cases by Dr. Mott.

A concise and well digested account of those parts of American surgery not contained in the original would have been much more valuable, and have occupied much less space than this accumulation of cases long since reported in the medical periodicals.

The plates of the Atlas embrace a great variety of instruments used in surgery, and are very well executed. D. B.

